# The Expression of Myeloproliferative Neoplasm-Associated Calreticulin Variants Depends on the Functionality of ER-Associated Degradation

**DOI:** 10.3390/cancers11121921

**Published:** 2019-12-02

**Authors:** Olivier Mansier, Valérie Prouzet-Mauléon, Gwénaële Jégou, Kim Barroso, Diana Pelizzari Raymundo, Aurélie Chauveau, Pierre-Yves Dumas, Valérie Lagarde, Béatrice Turcq, Jean-Max Pasquet, Jean-François Viallard, Chloé James, Vincent Praloran, Konstantinos Voutetakis, Aristotelis Chatziioannou, François-Xavier Mahon, Eric Chevet, Eric Lippert

**Affiliations:** 1 INSERM U1218, ACTION, Université de Bordeaux, UFR Sciences de la Vie et de la Santé, 33000 Bordeaux, France; olivier.mansier@u-bordeaux.fr (O.M.); Valerie.Prouzet-Mauleon@u-bordeaux.fr (V.P.-M.); valerie.lagarde@u-bordeaux.fr (V.L.); Beatrice.Turcq@u-bordeaux.fr (B.T.); francoisxavier.mahon@u-bordeaux.fr (F.-X.M.); 2 INSERM U1034, Université de Bordeaux, UFR Sciences de la Vie et de la Santé, 33600 Pessac, France; chloe.james@inserm.fr; 3 CHU Bordeaux, Laboratoire d’Hématologie, 33600 Pessac, France; vincent.praloran@u-bordeaux.fr; 4 INSERM U1242, Centre de Lutte Contre le Cancer, Eugène Marquis, Université de Rennes 1, 35042 Rennes, France; g.jegou@rennes.unicancer.fr (G.J.); baroyoupowa@hotmail.fr (K.B.); dianapelizzari@gmail.com (D.P.R.); 5 CHRU Brest, Laboratoire d’Hématologie, 29200 Brest, France; aurelie.chauveau@chu-brest.fr; 6 INSERM, EFS, UMR 1078, GGB, Université de Brest, 29200 Brest, France; 7 INSERM U1035, Université de Bordeaux, UFR Sciences de la Vie et de la Santé, 33000 Bordeaux, France; pierre-yves.dumas@u-bordeaux.fr (P.-Y.D.); jean-max.pasquet@u-bordeaux.fr (J.-M.P.); 8 CHU Bordeaux, Service d’Hématologie et Thérapie Cellulaire, 36000 Pessac, France; 9 CHU Bordeaux, Service de Médecine Interne, 36000 Pessac, France; jeanfrancois.viallard@chu-bordeaux.fr; 10 Institute of Chemical Biology, National Hellenic Research Foundation (N.H.R.F.), 116 35 Athens, Greece; kvoutet@eie.gr (K.V.); achatzi@eie.gr (A.C.); 11 Department of Biochemistry and Biotechnology, University of Thessaly, Viopolis, 41500 Larissa, Greece; 12 Institut Bergonié, 33000 Bordeaux, France

**Keywords:** calreticulin, ERAD, MPN, endoplasmic reticulum

## Abstract

Background: Mutations in *CALR* observed in myeloproliferative neoplasms (MPN) were recently shown to be pathogenic via their interaction with MPL and the subsequent activation of the Janus Kinase – Signal Transducer and Activator of Transcription (JAK-STAT) pathway. However, little is known on the impact of those variant CALR proteins on endoplasmic reticulum (ER) homeostasis. Methods: The impact of the expression of Wild Type (WT) or mutant CALR on ER homeostasis was assessed by quantifying the expression level of Unfolded Protein Response (UPR) target genes, splicing of X-box Binding Protein 1 (XBP1), and the expression level of endogenous lectins. Pharmacological and molecular (siRNA) screens were used to identify mechanisms involved in CALR mutant proteins degradation. Coimmunoprecipitations were performed to define more precisely actors involved in CALR proteins disposal. Results: We showed that the expression of CALR mutants alters neither ER homeostasis nor the sensitivity of hematopoietic cells towards ER stress-induced apoptosis. In contrast, the expression of CALR variants is generally low because of a combination of secretion and protein degradation mechanisms mostly mediated through the ER-Associated Degradation (ERAD)-proteasome pathway. Moreover, we identified a specific ERAD network involved in the degradation of CALR variants. Conclusions: We propose that this ERAD network could be considered as a potential therapeutic target for selectively inhibiting CALR mutant-dependent proliferation associated with MPN, and therefore attenuate the associated pathogenic outcomes.

## 1. Introduction

Essential Thrombocythaemia (ET) and Primary Myelofibrosis (PMF) are classical Philadelphia negative myeloproliferative neoplasms (MPN) characterized by a proliferation predominantly affecting the megakaryocytic compartment [1]. The pathophysiology of these diseases became clearer after the discovery of mutations in Janus Kinase 2 (JAK2) [2,3,4,5] and in the thrombopoietin receptor MPL [6] that were responsible for the constitutive activation of the Janus Kinase – Signal Transducer and Activator of Transcription (JAK-STAT) pathway. More recently, mutations in the calreticulin gene (*CALR*) bridged some gaps in our understanding of the oncogenesis of JAK2- and MPL-unmutated ET and PMF [7,8]. Calreticulin is an endoplasmic reticulum (ER) resident lectin, mainly involved in the quality control and productive folding of secretory glycoproteins [9]. Calreticulin, together with its transmembrane homolog calnexin [10] binds monoglucosylated, newly synthesized glycoproteins and contributes to their productive folding before export to their final destination site [11]. When newly synthesized glycoproteins cannot achieve proper folding due to the presence of mutations or environmental challenges, they enter a glycoprotein quality control machinery, comprising Uridine Diphosphate-glucose glucosyl transferases (UGGT) and ER mannosidases that will allow for more folding cycles [12]. Glycoproteins remaining improperly folded after these additional cycles are triggered for degradation through the ER Associated Degradation (ERAD)-proteasome system [13]. Misfolded proteins may accumulate in the ER, for instance, when ERAD is saturated, thereby leading to a condition known as ER stress [14]. ER stress triggers an adaptive signaling pathway named the Unfolded Protein Response (UPR) that aims at restoring ER homeostasis [15]. The UPR has been associated to different aspects of oncogenesis, mainly in solid tumors [16]. In MPN, the only available data concern Chronic Myeloid Leukemia (CML) in which UPR has been demonstrated as protective against cell mortality [17]. Thus far, however, there is scarce data documenting the role of the UPR or even the existence of an ER stress in non-CML MPN.

The *CALR* mutations discovered in ET and PMF consist of +1/−2 frameshift insertions/deletions in the exon 9, thereby leading to the generation of a novel positively charged C-terminal sequence with loss of the KDEL ER-retention motif [7,8]. Despite recent advances highlighting the interaction between mutant CALR and the MPL receptor to trigger the constitutive activation of the JAK-STAT pathway [18,19] the precise mechanisms involved in the oncogenic properties of those CALR variants remain poorly understood. Moreover, the subcellular localization and the fate of CALR variants remain controversial. Indeed, while some groups have detected these proteins mainly in the ER [7,8], others have suggested their preferential localization in the Golgi apparatus [18]. Besides, while the secretion of CALR mutant proteins has been suggested [20], other studies pointed towards a possible instability of these proteins and their subsequent degradation [18,21]. In order to develop therapeutic agents that specifically target the cells expressing CALR variant proteins, the determination of their biochemical properties appears to be instrumental. In the present study, we show that expression of CALR variants in various cell culture models does not significantly disturb ER homeostasis. To our surprise, CALR variants were found to be faintly expressed in cells, in part because of an excessive secretion associated with the loss of the KDEL motif, but also due to a catabolic process mainly mediated by the ERAD-proteasome system, thus allowing us to believe that MPN-associated CALR variants might indeed be considered as improperly folded proteins. To follow up on this, we identified specific components of the ERAD machinery that target the CALR variants and process them for degradation. Collectively, we show that CALR variants are genuine ERAD substrates and propose that modulations of ERAD activity could represent a therapeutic target for CALR-mutated MPN patients to attenuate CALR variant-dependent MPL activation.

## 2. Results

### 2.1. Mutant CALR are Faintly Expressed in Transfected and Primary Cells

**Figure 1:**
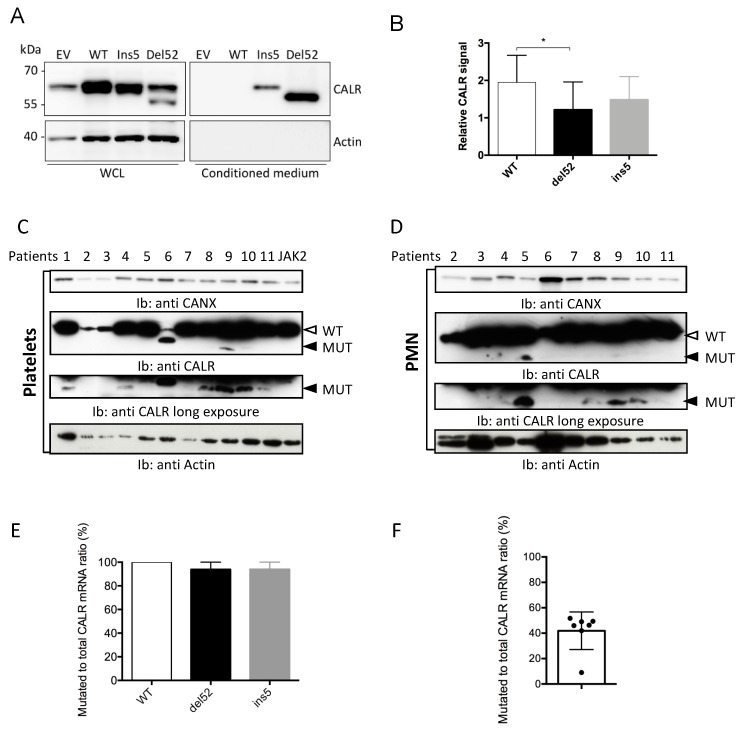
Calreticulin (CALR) mutant proteins are faintly expressed in the transfected and primary cells. (**A**) HEK293T cells were transfected with plasmids to express wild type (WT) or mutant CALR proteins. Forty-eight hours later, total cell lysates (TCL) and supernatant precipitates were subjected to western-blot analysis using anti-total CALR antibody. Actin serves as a loading control for cell lysates. (**B**) Quantification of signals measured on western-blot analysis of total cell lysates of transfected HEK293T cells performed in (A). Results are expressed as the ratio of CALR to actin signal normalized on the “Empty Vector” condition. The histogram represents the mean ± standard error of the mean (SEM) of six independent experiments. * *p* < 0.05 Platelets (**C**) and polymorphonuclear cells (PMN, (**D**) were purified from peripheral blood of 11 *CALR* mutated (patient numbers 1 to 11) and one *JAK2*V617F mutated MPN patients. CALR protein expression was assessed by western blotting using antitotal CALR antibody. Actin was used as loading control. The white arrowhead indicates WT CALR, the black one the position of the type-1 (del52) mutant CALR. All patients present a type 1 mutation (del52), except patient 6, who exhibits a homozygous type 2 (ins5) mutation. (**E**) Quantification of *CALR* mutant ratio on RNA of HEK293T 48 h after transfection. Results are expressed as the ratio of the copy numbers of mutant to total *CALR*. The histograms represent the mean +/− SEM of three independent experiments. (**F**) Quantification of *CALR* mutant ratio on RNA purified from peripheral PMN. Results are expressed as the ratio of the copy numbers of mutant to total *CALR*.

In order to study the properties of mutant CALR proteins, we constructed plasmidic vectors to express the wild type (WT) or the two most frequent mutant forms of CALR: type 1 (del52) or type 2 mutant (ins5). Unexpectedly, when these plasmids were transfected into HEK293T cells, we observed that mutant proteins were faintly expressed, as seen in Figure 1A,B. Indeed, upon transfection of the WT CALR encoding plasmid, a two-fold increase of total CALR protein level was observed, whereas this increase was only 1.2–1.5-fold after mutant allele expression. The low expression level of del52 was particularly obvious since this mutant protein presents a lower apparent molecular weight than the endogenous WT protein, as seen in Figure 1A,B. To make sure this was not an artifact due to transfection, mutant CALR protein level was studied in patients’ primary cells.

Western blots of platelets and polymorphonuclear cells purified from patients’ blood samples carrying CALR mutations confirmed a low level of mutant proteins. No difference in the expression level of mutant CALR proteins was observed between platelets and polymorphonuclear cells, suggesting that the presence of the MPL receptor did not affect the low expression level of CALR mutant proteins. In these two cell types, the difference of expression between WT and mutant CALR was even more pronounced than in transfected HEK293T cells, with del52 mutant protein being sometimes undetectable, despite high levels of WT protein, as seen in Figure 1C,D.

We thought of three main mechanisms to explain a lower expression level of variant CALR proteins: inefficient transcription/translation, protein degradation or, as these variant proteins are characterized by a loss of their KDEL ER-retention motif, increased leakage into the extracellular medium. A poor transcription rate was ruled out since CALR mutant mRNA were largely predominant when quantified in transfected HEK293T cells using digital droplet PCR (ddPCR), as seen in Figure 1E. Moreover, the ratio of mutated to total CALR mRNA determined in peripheral PMN was near 0.5 for most of the patients studied, as seen in Figure 1F. To address the question of the CALR secretion, proteins contained in the culture supernatants of transfected cells were precipitated with trichloroacetic acid (TCA) and analyzed by Western blot. We confirmed the detection of mutant CALR proteins in the extracellular medium while the WT protein was undetectable, thus confirming that a secretion process explained part of the low expression level of CALR variant proteins. 

### 2.2. Proteasome-Mediated Degradation Participates in Low CALR Mutant Expression

**Figure 2:**
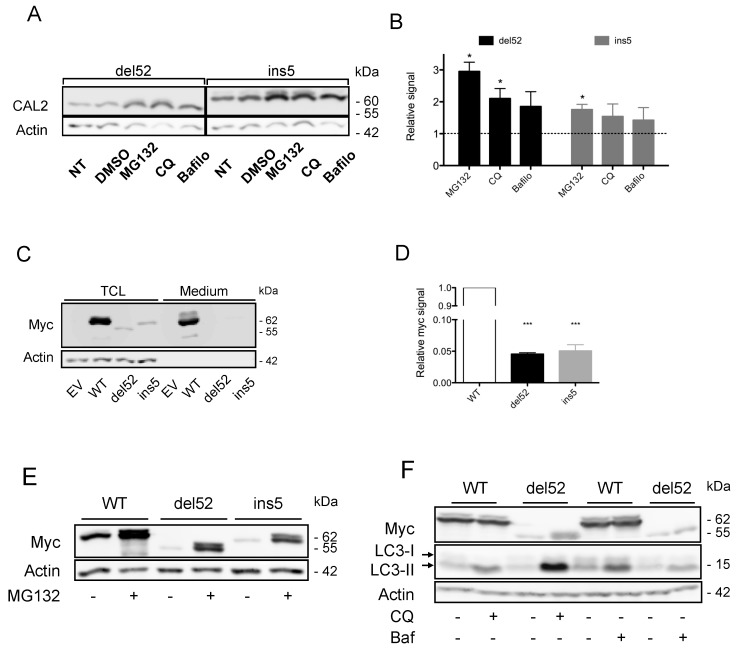
Calreticulin (CALR) mutant proteins are substrates of Endoplasmic Reticulum Associated Degradation (ERAD)-proteasome and autophagy degradation pathways. (**A**) HEK293T were transfected with plasmids to express non-tagged mutant CALR proteins. Twenty-four hours later, cells were treated by MG-132 (10 μM, Santa Cruz Biotechnology, Heidelberg, Germany), chloroquine (CQ, 50 μM, Sigma-Aldrich, Lyon, France), or Bafilomycin 1A (Bafilo, 100 nM, Sigma-Aldrich, Lyon, France) for another 24 h. The expression of the proteins was assessed by western blotting using the CAL2 antibody specific of the neoepitope generated by the frameshift mutations. Actin serves as a loading control. (**B**) Quantification results are expressed as a ratio of CAL2 signal to actin signal and then normalized against the “not treated” or “Dimethyl Sulfoxide” (DMSO) condition. Data are expressed as mean ± SEM from five experiments. * *p* < 0.05 (**C**) HEK293T were transfected with pcDNA6 plasmids encoding Myc-tagged WT or mutant CALR. Forty-eight hours later, total cell lysates (TCL) and supernatant precipitates were subjected to western blot analysis. Actin serves as a loading control for cell lysates. (**D**) Quantification of signals measured on western blot analysis of total cell lysates of transfected HEK293T cells performed in (C). Results are expressed as the ratio of Myc to actin signal, normalized on the CALR WT level. The histogram represents the mean ± SEM of three independent experiments. *** *p* < 0.001 (**E**,**F**) HEK293T were transfected with pcDNA6 plasmids to express Myc-6His tagged WT or mutant CALR proteins. Cells were treated 24h by (**E**) MG-132 (10 μM) or (**F**) chloroquine (CQ, 50 μM) or Bafilomycin A1 (Bafilo, 100 nM) and CALR expression was assessed by western-blotting with anti-Myc antibody. Actin was used as a loading control. Data presented are representative of three to ten experiments.

Secretion of mutated proteins is at least partly responsible for their low expression levels; however, since mutant to wild type ratios were even lower in primary cells, and because CALR is a central actor of the quality control system of glycoproteins in the secretory pathway, we wondered if excessive protein degradation could also partly explain the low expression level of CALR mutant proteins. CALR variants could therefore be considered misfolded proteins and would become substrates of the ERAD-proteasome system. To address this question, we treated transfected HEK293T cells with the proteasome inhibitor MG-132. This treatment increased CALR variants expression by mean factors of 2.9 and 1.8 for del52 and ins5 variants respectively (*p* = 0.014 and *p* = 0.0323), as seen in Figure 2A,B. 

These results indicate that both leakage to the extracellular medium and increased proteasome degradation participate in the reduction of mutant protein levels. The secretion mechanisms are relatively straight-forward, given the loss of the KDEL motif; however, the precise pathway leading to excess mutant CALR degradation has never been investigated. To study the processes controlling expression of CALR variants more precisely, we generated plasmids allowing the expression of Myc-6His-tagged CALR proteins, which could be readily revealed by Western blotting of their Myc epitope. The CALR proteins produced from these vectors are depicted in Figure S1A. After HEK293T cell transfection, WT CALR was expressed at high levels, whereas the expression of both mutants was very low (with an about 20-fold lower expression for del52 and ins5 mutants compared to the WT protein), as seen in Figure 2C,D. In contrast to “untagged” proteins, Myc-6-His-tagged CALR mutant proteins were not detected in the cell supernatant, as seen in Figure 2C, probably because the C-terminal part of the protein and thus the Myc-6His tag had been cleaved as previously suggested [22]. In these cells, the proteasome inhibitor MG-132 increased mutant CALR expression by a mean factor of five, as seen in Figure 2E.

To confirm that our results were not restricted to the “readily-transfectable” HEK293T cell model, we generated hematopoietic cell lines in which the expression of mutant (del52 and ins5), or WT CALR alleles was induced using tetracycline-inducible lentiviral vectors pCW57.1. In K562, DAMI, UT-7-MPL, and TF-1 cells, the expression levels of CALR variants were much lower than those observed for WT CALR; del52 mutant was almost undetectable and ins5 mutant was expressed at a level ~45-fold lower than the WT, as seen in Figure S1B,C. The mean mutated to total CALR mRNA ratio for del52 and ins5 mutants were, respectively, 62% and 61% in K562 cells, 60% and 55% in DAMI cells, 50% and 51% in UT-7 MPL cells, and 20% and 33% in TF-1 cells, as seen in Figure S1D, which represent values classically observed in *CALR* mutated MPN patients. Despite their low expression level, CALR mutant proteins, but not the WT protein, were able to trigger the activation of the JAK2-STAT5 signaling pathway, as seen in Figure S2A, and to support a Thrombopoietin (TPO)-independent growth in UT-7-MPL cells, as seen in Figure S2B. These results demonstrate that even if present in low quantity in the cells, CALR mutant proteins are able to induce a MPN phenotype as previously described [18,19]. In hematopoietic cells, MG-132 raised del52 and ins5 mutant expression by mean factors of 15 (*p* = 0.0091) and 12 (*p* = 0.0019), respectively, in UT-7 MPL and 7 (*p* = 0.0055) and 11 (*p* = 0.0051), respectively, in DAMI cells, as seen in Figure S3A–D. By contrast, this drug only increased the level of WT CALR by a mean factor of 1.7, 2.9, and 5.1 in HEK293T, DAMI, and UT-7-MPL cells, respectively. Of note, lactacystin, another proteasome inhibitor, increased mutant protein levels with an efficacy similar to that of MG-132.

With autophagy being a second possible mechanism involved in mutant CALR degradation, we inhibited this pathway by treating transfected or transduced cells with chloroquine and bafilomycin A1. In HEK293T, chloroquine and bafilomycin A1 were each able to block the autophagy process as assessed by the increase of LC3-II expression, as seen in Figure 2F. These treatments also increased expression of del52 mutant by a mean factor of 4.2 (*p* = 0.0022) and 1.6 (*p* = 0.0007), respectively, while they did not significantly increase WT CALR protein levels, as seen in Figure 2F. In hematopoietic cells, chloroquine and bafilomycin A1 only increased the expression levels of CALR ins5 mutant in DAMI cells by a mean factor of 2.8 (*p* = 0.0432) and 2.7 (*p* = 0.0177), respectively. No significant effect was observed for ins5 mutant in UT-7 MPL cells, nor for del52 mutant in DAMI and UT-7 MPL cells, despite efficient inhibition of the autophagy process as assessed by the increase of LC3-II expression level, as seen in Figure S3A–D. Of note, inhibition of autophagy had only limited effects on the level of expression of nontagged CALR proteins, as seen in Figure 2A,B. For the del52 variant, we observed an increased expression by mean factors of 2.1 and 1.9 with chloroquine and bafilomycin A1 treatments (*p* = 0.0397 and *p* = 0.14 respectively). No significant effect was observed on the level of ins5 mutant protein, neither for chloroquine, nor for bafilomycin A1.

Altogether, these experiments suggested that CALR mutant proteins were secreted, but that this phenomenon cannot explain by itself the low level of expression of these proteins. This leakage in the extracellular medium is combined to a degradation process involving mainly the ERAD-proteasome and, to a lower extent, the autophagy pathways. Finally, the inhibition of the proteasome restores expression of mutant proteins to levels similar to WT CALR protein in untreated cells.

### 2.3. Mutant CALR Expression Does Not Alter ER Homeostasis

**Figure 3:**
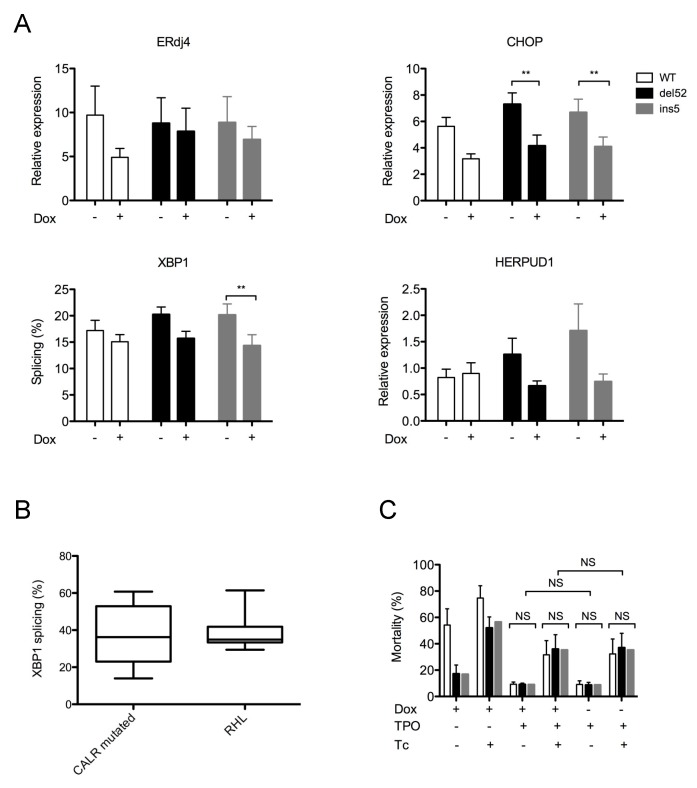
Impact of Calreticulin (CALR) mutant expression on endoplasmic reticulum (ER) homeostasis. Hematopoietic cell lines were transduced with pCW57.1 vector enabling tetracyclin-inducible expression of Myc-6His tagged wild type (WT) or mutant CALR. (**A**) The expression of Unfolded Protein Response (UPR) target genes was assessed in DAMI cells by RT-qPCR before and 48 h after treatment by doxycyclin (+Dox). GAPDH was used as control gene. The histograms represent the mean ± standard error of the mean (SEM) of three independent experiments. ** *p* < 0.01. (**B**) Leucocytes were obtained from peripheral blood samples of *CALR* mutated or patients presenting with reactive hyperleucocytosis and/or thrombocytosis (RHL). After extraction of RNA, XBP1 splicing was assessed by RT-PCR and densitometry analysis. The boxes represent the dispersion from minimum to maximum values. Ten patients were tested in each group. (**C**) Cell mortality induced by Tunicamycin (Tc, 10 μg/mL) was assessed by Annexin V- 4’,6-diamidino-2-phénylindole (DAPI) labeling in UT-7-MPL cells 24 h after CALR transgene expression induction by Doxycyclin (Dox). These experiments were performed in the presence or in the absence of thrombopoietin (TPO). Dimethyl Sulfoxide (DMSO) was used as a vehicle control. Results are expressed as the mean proportion of cells positive for Annexin V in four independent experiments.

Because CALR mutants seem to be the substrates of the ERAD-proteasome pathway, we asked whether the expression of CALR mutants disturbed ER homeostasis. Because megakaryocytes are central actors in the pathogenesis of ET and PMF, we focused on two cell lines with megakaryocytic features (UT-7 and DAMI) to address this question. Alteration of the three main branches of UPR was assessed by quantification of representative target genes (*Erdj4*, *CHOP*, *HERPUD1*) and splicing of *XBP1*. In the DAMI cell line, neither the induction of del52, nor that of ins5 CALR variants significantly induced the expression of these ER stress markers, but on the contrary, led to a global decrease in the expression of UPR target genes, as seen in Figure 3A. This was statistically significant for XBP1 mRNA splicing (for type 2 mutant, *p* = 0.0055) and CHOP mRNA expression (for both del52 and ins5 mutants, *p* = 0.0098 and *p* = 0.0061, respectively). Similar results were observed in UT-7-MPL cells. Of note, overexpression of WT CALR showed the same trend at reducing UPR target gene expression, in particular for Erdj4 and CHOP, even if not statistically significant. Accordingly, ER stress determined by XBP1 splicing was not different between *CALR* mutated patients and control patients presenting with reactive thrombocytosis and/or hyperleukocytosis, as seen in Figure 3B, confirming that CALR mutant expression does not disturb ER homeostasis. Because CALR variants could alter the consequences of an ER stress rather than generating it, we sought to determine whether the expression of CALR mutants could alter ER stress-induced apoptosis. A 24 h or 48h exposure of UT-7-MPL cells to tunicamycin (10 μg/mL), as seen in Figure 3C, or MG-132 increased cell mortality in the absence, as well as in the presence of TPO. In all conditions, tunicamycin induced apoptosis to the same extent, whether cells expressed WT or mutant calreticulin. Since CALR mutations are rarely homozygous in MPN cells, as opposed to other driver mutations such as *JAK2*V617F or *MPL*W515, it can be hypothesized that normal CALR in the heterozygous cells appears sufficient to maintain cell homeostasis. 

**Figure 4:**
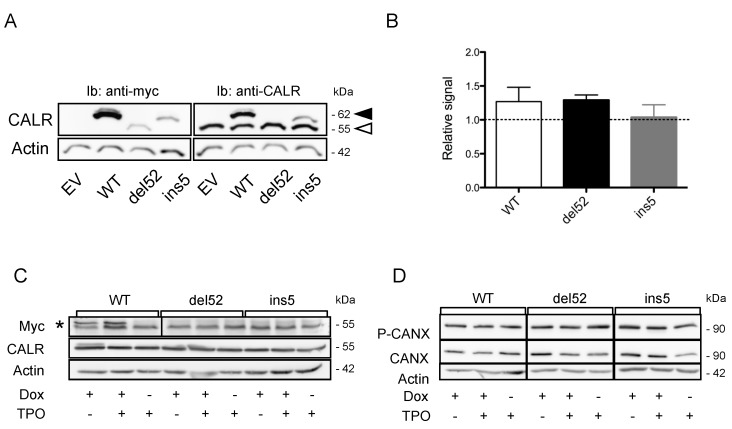
Expression of Calreticulin (CALR) mutants does not alter the expression of endogenous CALR protein. (**A**) HEK293T were transfected with pcDNA6 plasmids to express Myc-6His tagged wild type (WT) or mutant CALR proteins. Forty-eight hours later, endogenous WT CALR expression was assessed by western blotting using the antitotal CALR antibody. Actin was used as a loading control. Note that because of the presence of the tag, the CALR proteins present with an increased apparent molecular weight (MW) compared to the nontagged forms. Hence, WT and ins5 proteins (black arrowhead) present a MW superior to the endogenous nontagged WT CALR protein (white arrowhead), while the tagged form of del52 protein (the Molecular Weight of which is lower than that of WT and ins5) present the same MW as the WT endogenous protein. (**B**) The expression of endogenous CALR was quantified and expressed as the ratio of CALR to actin signal and normalized against the “empty vector” (EV) condition. For Myc-6His tagged del52 mutant CALR (which presents the same MW as the endogenous CALR) a correction was applied by subtracting the signal generated by the transgene estimated from the comparison of the signal measured for WT and ins5 CALR with anti-Myc and anti-CALR antibodies. Results are presented as mean ± standard error of the mean (SEM) of three independent experiments. (**C**) UT-7 cells were transduced with pCW57.1 vector enabling tetracyclin-inducible expression of Myc-6His tagged WT or mutant *CALR*. Expression of WT, type 1 (del52), and type 2 (ins5) *CALR* mutants was induced by Doxycyclin treatment. Forty-eight hours later, endogenous WT CALR expression was assessed by western blotting using the antitotal CALR antibody. Actin was used as a loading control. For Myc labeling, * denotes the specific signal, as determined by the molecular weight. (**D**) Expression of WT, type 1 (del52), and type 2 (ins5) CALR mutants was induced in UT-7 cells using a tetracyclin-inducible vector. Forty-eight hours later, Calnexin (CANX) expression and phosphorylation were assessed by western blotting. Actin was used as a loading control.

The absence of increased ER stress by CALR mutant expression could be due to a compensatory overexpression of CALR WT. To address this question, we transfected HEK293T cells with CALR WT or mutant vectors. Endogenous CALR expression was not modified by mutant CALR protein expression, as seen in Figure 4A,B. Similarly, induction of CALR mutants in UT-7 cells transduced with the pCW57.1 tetracyclin-inducible vector did not modify endogenous CALR expression, as seen in Figure 4C. Because the quality control system in the secretory pathway involves another lectin than CALR (calnexin or CANX), we sought to analyze CANX expression in the presence or absence of CALR variants. As observed for endogenous CALR, CANX expression level was not modified by CALR mutants. CANX phosphorylation, which may reflect its stress-regulated functions [23,24], was not modified either, as seen in Figure 4D. Altogether, these results suggest that expression of CALR mutants does not significantly disturb ER homeostasis and does not impact cell sensitivity toward ER stress-induced apoptosis. Hence, CALR mutant-associated alteration of ER function does not appear to represent a significant mechanism in MPN development associated with *CALR* mutations.

### 2.4. ERAD-Dependent Mechanisms of CALR Variants Degradation

To get further insight into the molecular mechanisms involved in the degradation of CALR mutant proteins, we treated CALR expressing HEK293T with different drugs targeting the ER quality control (ERQC), ERAD pathway, and other degradation pathways. 

**Figure 5:**
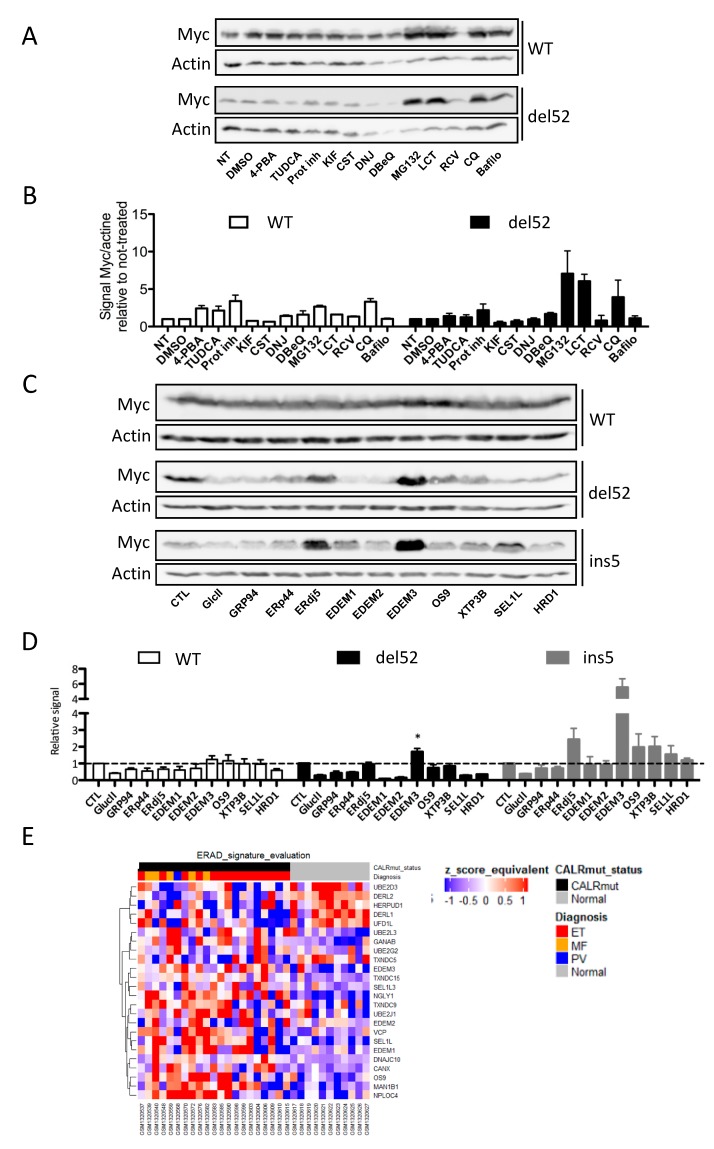
Endoplasmic reticulum Degradation-Enhancing α-Mannosidase-like protein 3 (EDEM3) is a key player for targeting Calreticulin (CALR) mutants to the Endoplasmic Reticulum Associated Degradation (ERAD)-proteasome pathway. (**A**) HEK293T were transfected with pcDNA6 plasmids encoding Myc-6His tagged wild type (WT) or mutant CALR proteins. Twenty-four hours post transfection, the cells were treated with the indicated pharmacological agents at the concentration indicated in the Materials and Methods section. CALR expression was then assessed by western blotting using anti-Myc antibody. Actin serves as a loading control. Each lane corresponds to a drug as indicated in (B) and with the same order. (**B**) The expression of WT and type 1 (del52) mutant of CALR was assessed and expressed as the ratio of Myc signal to actin signal normalized against the “not treated” or “Dimethyl Sulfoxide” (DMSO) condition. The results presented represent mean +/− standard error of the mean (SEM) determined in two independent experiments. (**C**) HEK293T cells were transfected with siRNA directed against different actors of the ERAD pathway. Forty-eight hours later, they were transfected with pcDNA6-CALR plasmids. Twenty-four hours later, cells were lysed before analysis by western blotting. CALR proteins were detected via their Myc epitope and actin was used as a loading control. Each lane corresponds to a siRNA as indicated in (D) and with the same order. (**D**) The expression of the different forms of CALR secondary to ERAD actors silencing was quantified as in (B). The histograms represent mean ± SEM determined in three to six independent experiments. * *p* < 0.05 (**E**) Hierarchical clustering was performed on gene expression microarray data from Rampal et al. [25]. The expression of Endoplasmic Reticulum (ER) quality control and ERAD actors in *CALR* mutated Myeloproliferative Neoplasm (MPN) patients was compared to matched normal patients. Blue denotes decreased expression, while red represents increased expression. NT: Not treated, 4-PBA: 4-phenylbutyrate, TUDCA: Tauroursodesoxycholic acid, Prot inh: Protease inhibitors, KIF: Kifunensin, CST: Castanospermin, DNJ: 1-Deoxynojirimycin, LCT: Lactacystin, RCV: Roscovitin, CQ: Chloroquine, Bafilo: Bafilomycin A1. DMSO was used as a control for all drugs except for 4-PBA, protease inhibitors, and chloroquine.

To determine if an alternative degradation pathway involving the lysosomes was involved in CALR proteins degradation, we treated transfected HEK293T cells with a protease inhibitor cocktail (COmplete, Roche Diagnostics, Meylan, France), as seen in Figure 5A,B, but did not observe any significant increase in their expression. Moreover, we raised the hypothesis that mutant CALR could behave as partially misfolded proteins subjected to quality control mechanisms. To test this hypothesis, we monitored the impact on CALR mutant expression of TUDCA and 4-PBA, two molecules shown to increase the ER folding capacity and decrease the misfolded protein burden [19]. Interestingly, neither TUDCA nor 4-PBA had any significant effect, as seen in Figure 5A,B, suggesting that CALR variants may behave/be recognized as terminally misfolded proteins. 

Regarding actors of the ERAD pathway, we did not observe any effect of the glucosidase inhibitors (Castanospermin, 1-Deoxynojirimycin), the mannosidase inhibitor (Kifunensin), the p97 inhibitor DBeQ, or Roscovitin (previously shown to increase the expression of the ERAD-substrate CFTR Δ508 [25]), as seen in Figure 5A,B. These results suggested that recognition of a glycan motif was not involved in the recognition of mutant CALR proteins as a substrate for the ERAD pathway and that the proteasome-mediated degradation may not directly require the AAA+ ATPase p97. To further characterize the ERAD-dependent intermediates involved in the degradation of CALR variants, we carried out an ERQC/ERAD targeted siRNA screen in HEK293T cells and evaluated the potential stabilization of del52 and ins5 CALR mutants. Among the candidates tested, EDEM3 was identified as a key player in the degradation of both CALR mutants, whereas ERdj5/DNAJC10 appeared to be a relevant player specifically for the ins5 mutant, as seen in Figure 5C,D. Indeed, EDEM3 silencing led to a 1.7-fold (*p* = 0.0278) and a 5.5-fold (*p* = 0.0171) increase in the expression of the del52 and ins5 mutants, respectively. The effect of EDEM3 silencing on WT CALR was lower, with a mean increase of 1.5-fold (*p* = 0.05). In contrast, silencing of EDEM1 and EDEM2, other members of the Endoplasmic reticulum Degradation-Enhancing α-Mannosidase-like protein (EDEM) family, had no impact on mutant CALR proteins expression. Of note, glucosidase II silencing did not increase CALR mutant expression levels, thereby confirming that recognition of these proteins as ERAD-substrate was glycan-independent. Finally, siRNA targeting ER chaperon (GRP94), lectins involved in transfer of ERAD-substrate to the retrotranslocon (OS-9, XTP3-B) or components of the retrotranslocon itself (SEL1L, HRD1) did not increase significantly the expression level of mutant or WT-CALR. 

In order to test whether the ERAD machinery might be affected in *CALR*-mutated MPN patients, we analyzed the expression of select ERAD genes in the dataset generated by Rampal et al. [26]. In accordance with the mechanism of CALR mutant degradation, we observed a significant deregulation of the expression of different ERAD components such as EDEM3, CANX, DNAJC10, SEL1L, VCP and different components of ubiquitin ligase complex in MPN samples, as seen in Figure 5E.

### 2.5. Identification of a Network Involved in the ERAD-Dependent Degradation of CALR Variants

**Figure 6:**
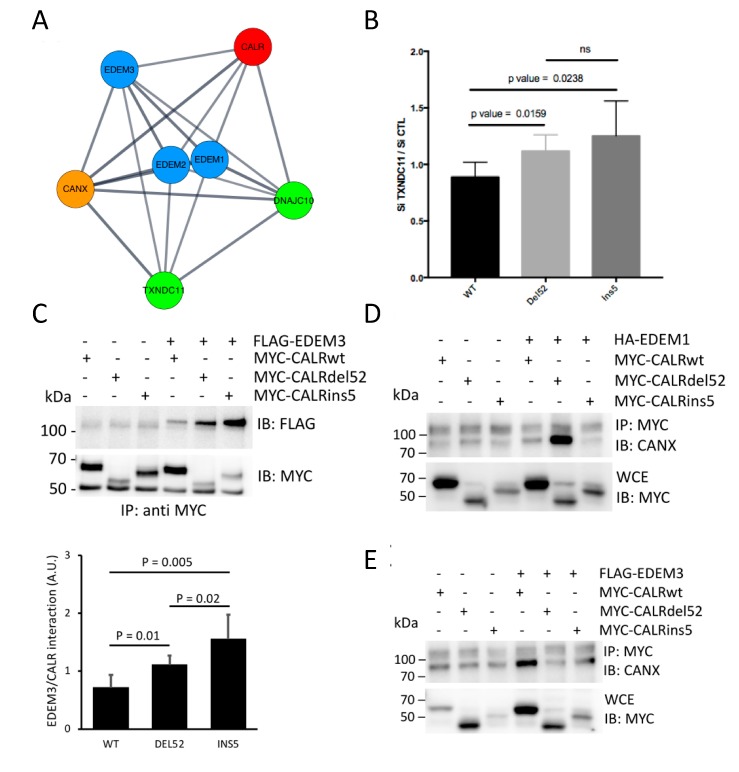
Calreticulin (CALR) mutant proteins are subjected to an interaction with a network involving different proximal Endoplasmic Reticulum Associated Degradation (ERAD) actors. (**A**) Graphical representation of the actors involved in a network leading to the disposal of mutant CALR proteins based on results observed in Figure 5, available databases (https://string-db.org/; https://thebiogrid.org/; https://www.ebi.ac.uk/intact/), and literature. (**B**) HEK293T cells were transfected with siRNA directed against TXNDC11 or a scramble control. Twenty-four hours later, cells were transfected with pcDNA6-CALR plasmids. Twenty-four hours later, cells were lysed before analysis by western blotting. CALR proteins were detected via their Myc epitope and actin was used as a loading control. The expression of the different forms of CALR secondary to TXNDC11 silencing was expressed as in Figure 5D. The histograms represent mean ± standard error of the mean (SEM) determined in four independent experiments. (**C**) Upper panel: HEK293T were cotransfected with pcDNA6 plasmids to express Myc-6His tagged wild type (WT) or mutant CALR proteins together with a plasmid allowing the expression of FLAG-tagged Endoplasmic reticulum Degradation-Enhancing α-Mannosidase-like protein 3 (EDEM3) protein. Cell lysates were subjected to immunoprecipitation with an anti-Myc antibody and then subjected to western-blot analysis. IP: Immunoprecipitation, IB: Immunoblot. Lower panel: Interaction of CALR with EDEM3 was quantified as the ratio of EDEM3 and CALR A.U.: Arbitrary Unit. (**D**,**E**) HEK293T were cotransfected with pcDNA6 plasmids to express Myc-6His tagged WT or mutant CALR proteins together with a plasmid allowing the expression of FLAG-tagged EDEM3 (**D**) or HA-tagged EDEM1 (**E**) proteins. Cell lysates were subjected to immunoprecipitation with an anti-Myc antibody and then subjected to western-blot analysis using an anticalnexin (CANX) antibody. Shown are representative results observed in three independent experiments. IP: Immunoprecipitation, IB: Immunoblot, WCE: Whole cell extracts.

We then collated all the information gathered in our siRNA screen, the available databases, and the literature. This identified a CALR closed network containing the EDEM proteins, as seen in Figure 6A, blue nodes, the CALR homolog CANX, as seen in Figure 6A, orange node, and the thioredoxin domain containing proteins DNAJC10 and TXNDC11 [27], as seen in Figure 6A, green nodes. Based on this, we first tested whether siRNA-mediated silencing of TXNDC11 impacted on the expression of CALR mutants. This showed that TXNDC11 expression attenuation led to a stabilization of both del52 and ins5 variants, as seen in Figure 6B. As such, together with the results obtained in Figure 5, this shows that specific thioredoxin domain containing proteins might be involved in the degradation of CALR variants, with a preferential impact on the ins5 mutant. Since EDEM3 silencing was found to stabilize both del52 and ins5 variants, we then tested whether these proteins could be found in a complex. Coimmunoprecipitations studies showed that the del52 and ins5 variants were more prone to be found in EDEM3-containing complexes than the WT CALR, as seen in Figure 6C. To follow up on the CALR network identified in Figure 6A, we monitored the presence of complexes comprising both CANX and CALR, and evaluated whether the expression of EDEM1 or EDEM3 might impact on those complexes. This showed that overexpression of EDEM1 selectively stabilized a del52 CALR/CANX complex, as seen in Figure 6D, whereas the overexpression of EDEM3 did not significantly impact on the formation of mutant CALR/CANX complexes, but increased the WT CALR/CANX complex, as seen in Figure 6E. These results show that the expression of CALR variants is dependent on a proximal ER quality control system, most likely including EDEM3, TXNDC11, and DNAJC10, which, when deregulated in MPN, might lead to sufficient stabilization of the proteins to yield constitutive MPL activation.

## 3. Discussion

In this study, we have determined the impact of the expression of CALR variants on the ER homeostasis and investigated the underlying mechanisms involved in their observed low expression level. We showed that expression of CALR mutant proteins associated with MPN does not trigger an ER stress. These results were unexpected given that it was recently deduced from transcriptomic data that ER homeostasis was perturbed in CALR variant-expressing MPN [28,29]. However, our data suggest that this UPR pathway activation is not a direct consequence of the expression of CALR mutant proteins. On the contrary, it might result from the modification of cell properties, such as an increased proliferation, as suggested by a more predominant activation of the UPR in committed progenitors compared to hematopoietic stem cells (HSC) [29]. Salati et al. recently reported a decreased sensitivity toward ER-stress induced apoptosis in cells expressing CALR variants [30]. However, these results were obtained in K562 cells that do not express MPL and the proliferation of which is not dependent on cytokines. In contrast, we observed in two different cell lines with megakaryocytic features (including MPL expression) that cells expressing the CALR variant proteins are as sensitive to ER stress-induced apoptosis as cells expressing the WT CALR, which is consistent with the absence of benefit of proteasome inhibitor bortezomib in the treatment of PMF patients, a molecule known to trigger cell apoptosis through induction of an ER-stress [31,32]. Taken together, these results suggest that activation of ER adaptive signaling may not be a major determinant of the pathophysiology of *CALR* mutated-MPN oncogenesis. However, we cannot rule out an involvement of ER stress in different aspects not assessed in this study or restricted to specific cell populations. Of note, the ER stress in HSC has been associated with a priming toward megakaryocytic lineage [29]. Thus, the activation of the UPR in HSC could result in a preferential differentiation of HSC into megakaryocytes, as previously suggested [33]. 

The low expression level of CALR mutant proteins compared to their WT counterpart was previously reported in numerous studies [18,19,21,22], but the mechanisms involved in this phenomenon have generally not been addressed. Other groups have reported a secretion of CALR variant proteins in the extracellular medium [20,22,34,35]. In these studies, as in ours, CALR variants were detectable in the extracellular medium while the WT form was absent, as seen in Figure 1A. However, the amount of mutant proteins in the cell supernatants seemed too low to explain the difference of expression level between WT and mutant proteins in the cell lysates. Moreover, Sollazzo et al. observed a similar level of plasmatic CALR between *JAK2*- and *CALR*-mutated MPN patients [36], suggesting that the secretion of mutant CALR proteins is not massive. Accordingly, when using the Myc-6His tagged forms of CALR, we detected significant amounts of WT CALR in the extracellular medium, probably because the Myc-6His tag prevented the ER-retention by the KDEL motif. However, this leakage was not sufficient to significantly decrease the expression level of CALR WT protein. This prompted us to search for other mechanisms that would be combined to the secretion of mutant CALR proteins to explain their faint expression. Interestingly the treatment of various cell lines with two different proteasome inhibitors (MG-132 and Lactacystin) revealed that these variant proteins were stabilized, and thus that they may be subjected to degradation via the ERAD-proteasome pathway. This process is not the consequence of the presence of a tag on the C-terminal end of the proteins, since 1) increased level of CALR variants was observed after MG132 treatment in another study using N-terminal tagged forms of CALR [21]; 2) we confirmed a low expression level of endogenous mutant CALR in primary patients’ cells; and 3) a similar degradation process was monitored after transfection of nontagged mutant proteins in HEK293T, as seen in Figure 2A,B. Inhibition of autophagy showed moderate effects on mutant CALR proteins’ expression and discrepancies were noticed depending on the drug and the cell line. Moreover, we did not notice a robust and reproducible additive effect of autophagy and proteasome inhibition over proteasome inhibition alone, suggesting a limited role of autophagy in CALR variants’ clearance. In these conditions, one might postulate that the contribution of autophagy could be restricted to situations where the ERAD pathway is saturated, as previously shown in other models [37]. To the best of our knowledge, the CALR variants are the first example of oncogenic proteins subjected to a major degradation mechanism. This phenomenon could be due to a toxic gain-of-function of the mutants when present in excess amount. This is supported by the fact that *CALR* mutations are exceptionally homozygous [7]. Moreover, the generation of *CALR* mutations by the CRISPR technology in conditions of cytokine deprivation results in predominantly heterozygous mutations [38]. As a consequence, it can be hypothesized that the gain of pro-oncogenic functions associated with CALR variants must occur at concentrations of the variants that exhibit low toxicity and thus might be extremely dependent on the mechanisms controlling the expression level of those variants.

The features allowing the recognition of CALR variants as ERAD substrates are not known. Mammalian CALR protein presents a single N-glycosylation site, but an effective glycosylation is detected only under stress conditions [39]. Therefore, it is not surprising that glycan motifs may not be involved in the degradation process of CALR variants as suggested by the absence of effect of glucosidase and mannosidase inhibition (using both pharmacological and genetic tools). However, it can be hypothesized that the recognition involves the C-terminal sequence common to all CALR variants and/or the conformational changes this might induce. Indeed, the degradation mechanisms are observed for both the “long deletion” (such as del52) and small insertion (such as ins5) variants. Moreover, the deletion of all or a part of the last 36 amino-acids results in an increased expression of the variant proteins [22,40]. The siRNA screen carried out in this study showed that CALR variant degradation does not involve the classical ERAD machinery for soluble ER luminal glycoproteins (i.e., OS-9, XTP3-B, SEL1L, HRD1). Moreover, DBeQ failed at increasing expression levels of mutated CALR suggesting that the AAA+ ATPase p97 may not be an actor of CALR variants disposal, even though involved in the degradation of a large variety of ERAD substrates [41]. Nevertheless, we highlighted EDEM3 as a key component in mutant CALR degradation pathway. Moreover, the absence of effect of kifunensine treatment suggests that EDEM-associated potential mannosidase activity is dispensable for this process. Concordantly, the knock-down of EDEM2 acting upstream of EDEM3 in mannose chain trimming [42] has no effect on the expression level of del52 and ins5 mutants. EDEM3, like other members of the EDEM family, has previously been shown to interact with nonglycosylated substrates [43]. However, its role in the degradation of proteins has been limited to glycosylated substrates thus far. The identity of possible other factors involved in CALR mutated protein disposal remains to be determined, but our observations confirm the existence of a specific equipment dedicated to the degradation of nonglycosylated substrates, as previously suggested [44]. 

Interestingly, we found that the expression of ERAD genes was selectively altered in *CALR* variants expressing MPN using existing datasets [26], thus confirming a pathophysiological link between ERAD and CALR variants. This concept was reinforced by the presence of complexes containing CALR variants and selective ER quality control and ERAD components. A recent study also described an increased interaction of mutant CALR proteins with Endoplasmic Reticulum Quality Control (ERQC) actors [45]. As such, one might propose a model in which the combined expression of CALR variants and an altered ERAD network might concur with the development of a myeloproliferative phenotype. In such a model, a strategy aiming at restoring the properties of the altered ERAD pathway might in turn promote the degradation of CALR variants and decrease the pro-oncogenic burden. In particular, modulating the expression of EDEM proteins influences the binding of CALR proteins to CANX. Because the release of ERAD substrates from CANX represents the first step of the degradation process, disturbing the balance in the EDEM protein family could impact the clearance of the CALR mutant proteins. At present, the specific deregulation of ERAD in MPN needs to be further characterized in order to identify the pharmacological compounds susceptible to being active in such a context. Because such a strategy has shown benefits in neurodegenerative diseases [46,47], modulating the degradation of CALR mutant proteins by the ERAD-proteasome pathway might represent an appealing strategy to improve the management of *CALR*-mutated MPN.

## 4. Materials and Methods 

### 4.1. Patients and DNA Constructs 

Complementary DNA from patients with *CALR* WT, type 1 (del52, NM_004343:c.1099_1150del) or type 2 (ins5, c.1154_1155insTTGTC) mutants were obtained at CRB-Cancer—CHU de Bordeaux. They were cloned into pcDNA6-Myc-6His. For the WT CALR, the tag was introduced after the KDEL sequence. Tagged CALR cDNA was then cloned into the pCW57.1 (tetracycline-inducible expression; kind gift of R. Iggo, Univ. Bordeaux) using the Gateway technology (Thermo Fisher Scientific, Illkirch, France). Alternatively, nontagged WT, del52, and ins5 CALR were obtained by directly cloning their cDNA into a lentiviral vector bearing a GFP reporter gene. The MPL cDNA was obtained from a healthy donor and cloned into a lentiviral vector carrying a Tomato fluorescent reporter. EDEM3 cDNA was amplified from HEK293T cells and cloned into a lentiviral vector carrying a ZsGreen fluorescent reporter with insertion of a 3X-Flag tag before the KDEL motif. cDNA of MPN patients and controls with reactive hyperleukocytosis and/or thrombocytosis were obtained from peripheral leukocyte samples after lysis of red blood cells and purification with QIAmp Blood mini kit (Qiagen, Courtaboeuf, France). All patients provided written informed consent for the use of remnant DNA for investigational purposes in accordance with the declaration of Helsinki. The HA-EDEM1 construct was described previously [48]. This study has been approved on the 9th April 2015 by the ethics committee of the university hospital of Bordeaux (ref DC2015/52).

### 4.2. Cell Culture and Lentiviral Transduction

HEK293T cells were cultured in DMEM (Life technology, Courtaboeuf, France) supplemented with 10% Fetal Bovine Serum (FBS), penicillin 100 U/mL, streptomycin 100 μg/mL, and L-glutamine 2 mM and were transfected using the calcium phosphate method or with polyethylenimine reagent (Sigma-Aldrich, Lyon, France). K562 and TF-1 were obtained from DSMZ and cultured in Roswell Park Memorial Institute medium (RPMI, Life technology, Courtaboeuf, France) supplemented with 10% FBS, penicillin 100 U/mL, streptomycin 100 μg/mL, and L-glutamine 2 mM. UT-7 MPL were generated by transduction of UT-7 EPO cells with a MPL lentiviral vector and cultured in IMDM (GE Healthcare, Chicago, IL, USA; Fisher Scientific, Illkirch, France) supplemented with 10% FBS, penicillin 100 U/mL, streptomycin 100 μg/mL, L-glutamine 2 mM, 4-(2-hydroxyethyl)-1-piperazineethanesulfonic acid (HEPES) 10mM, and β-mercaptoethanol 50 μM. TF-1 and UT-7 MPL were cultured in presence of 10 ng/mL of rhIL3 or rhTPO respectively. DAMI cells were cultured in IMDM supplemented with 10% FBS, penicillin 100 U/mL, streptomycin 100 μg/mL, and L-glutamine 2 mM. None of these cell lines present a mutation in the *JAK2* gene. Lentiviral vectors were produced by the vectorology platform of the University of Bordeaux. After transduction, cells were selected by puromycin resistance or sorted on the expression of the fluorescent reporter. Expression of the transgenes in pCW57.1 was induced by treatment with 2 μg/mL doxycycline (Sigma-Aldrich, Lyon, France).

### 4.3. Chemicals and Antibodies

Proteasome inhibitor MG-132 (10 μM) and DBeQ (5 μM) were obtained from Santa Cruz Biotechnologies (Heidelberg, Germany). Autophagy inhibitors chloroquine (CQ, 50–100 μM) and Bafilomycin A1 (Bafilo, 100 μM), Tauroursodesoxycholic acid (TUDCA, 1 mM) and 4-phenylbutyric acid (4-PBA; 5 mM), the glucosidase inhibitor castanospermin (CST, 750 μM) and the mannosidase inhibitor kifunensin (KIF, 25 μg/mL) were from Sigma Aldrich (Lyon, France). The proteasome inhibitor lactacystin (LCT, 50 μM), the glucosidase inhibitor 1-Deoxynojirimycin (DNJ, 1 mM), and the N-glycosylation inhibitor tunicamycin (Tc, 10μg/mL) were obtained from Calbiochem (Fontenay sous Bois, France). COmplete protease inhibitor was from Roche Diagnostics (Meylan, France). Monoclonal rabbit anti- calreticulin (EPR3924) and rabbit polyclonal anti-MPL (06-944) were from Merck Millipore (Fontenay sous bois, France). The mouse monoclonal antibody CAL2 against the novel C-terminal sequence of the CALR mutants was purchased from Dianova. The mouse monoclonal anti-Myc antibody (9E10) and rabbit anti-STAT5 were ordered from Santa Cruz Biotechnology (Heidelberg, Germany). Monoclonal rabbit antiactin antibody was from Sigma-Aldrich (Lyon, France). Monoclonal antibody against Flag tag (F1804) was from Sigma-Aldrich (Lyon, France). Rabbit anti-Phospho-STAT5-antibody (Y694) was purchased from Cell Signaling Technology (Leiden, Netherlands). Annexin V-APC was from eBiosciences (Thermo Fisher Scientific, Illkirch, France) and 4’,6-diamidino-2-phénylindole (DAPI) from Thermo Fisher Scientific (Illkirch, France). Both anticalnexin and antiphosphocalnexin antibodies were previously described and kindly provided by Dr. John Bergeron (McGill University, Montreal, QC, Canada) [24].

### 4.4. RNA Interference

siRNAs against different actors of ERAD were obtained from Ambion (ThermoFischer Scientific) or from a previously described siRNA library [17]. These oligonucleotides were delivered into HEK293T cells by transfection with calcium phosphate or polyethylenimine reagent at a concentration of 50 nM.

### 4.5. Coimmunoprecipitation and Western Blotting

Cell lysates obtained following lysis with 20 mM Tris-HCl pH 7.5, 150 mM NaCl, 1.5% CHAPS, protease inhibitor cocktail (Complete) were clarified by centrifugation. Immunoprecipitating anti-Myc antibodies were incubated overnight and collected using protein G-coupled magnetic beads washed extensively using lysis buffer and resuspended in Laemmli sample buffer. Immunoprecipitates or cell lysates resuspended in Laemmli buffer or Cell Lysis Buffer (Cell Signalling Technology, Danvers, MA, USA) were resolved by Sodium Dodecyl Sulfate Polyacrylamide Gel Electrophoresis (SDS-PAGE) in order to load equal quantity of proteins diluted in the same volume. Proteins were then transferred to PolyVinyliDene Fluoride (PVDF) membranes using the Transblot Turbo system (Bio-Rad Laboratories, Inc., Hercules, CA, USA). Membranes were incubated with the indicated primary antibodies and appropriate Horseradish Peroxidase (HRP)-coupled secondary antibodies. Immunoreactive proteins were detected via chemoluminescence with a LAS-3000 Camera (Fujifilm, Tokyo, Japan) or Amersham Hyperfilm MP autoradiography films (GE Healthcare, Chicago, IL, USA). When quantification was performed (using ImageJ software), only data obtained with the camera were used, allowing a larger linear range and the detection of saturated signal. Secreted proteins were precipitated from conditioned media with trichloroacetic acid (10% v/v). The pellets were then washed twice with 80% acetone and resuspended in Laemmli buffer before analysis by Western blot.

### 4.6. RT-qPCR and ddPCR

Total RNA was extracted using Trizol reagent (Life technologies, Courtaboeuf, France), treated by DNAse (Ambion) and reverse-transcribed with First Strand cDNA synthesis kit (Roche Diagnostics, Meylan, France). CALR mRNA expression was assessed by digital droplets PCR (ddPCR) as previously described [49]. UPR target gene expression was evaluated by RT-qPCR as previously described [50].

### 4.7. Gene Expression Microarray Analysis and Statistical Selection

Affymetrix raw microarray data of 93 patients with MPNs (28 PV, 47 ET, 18 MF) and 11 age-matched normal donors, deposited in Gene Expression Omnibus (GEO) database with the accession number of GSE54644, were retrieved and their gene expression values were background corrected, log2 transformed, and then quantile normalized using Robust Multiarray Averaging (RMA). Linear Models for Microarray Data (LIMMA) was applied to identify any differentially expressed genes between CALR mutants (21 samples) and their matched normal samples. Differentially expressed genes with *p*-value ≤ 0.05 and False Discovery Rate (FDR) ≤ 0.1 were highlighted as statistically significant. Microarray, differential expression and exploratory data analysis were performed using the open-source R/Bioconductor programming environment.

### 4.8. Statistical Analyses

Statistical analysis was performed by one-way ANOVA, paired *t*-test (to evaluate the impact of treatments), or unpaired *t*-test (to compare the effects of the different forms of CALR). All analyses were performed using Prism software (GraphPad, San Diego, CA, USA). Significant differences were determined by *p* < 0.05.

## 5. Conclusions

The expression of CALR mutant proteins does not significantly disturb ER homeostasis, nor impact ER stress-induced apoptosis. These proteins are faintly expressed due to a secretion into the extracellular medium combined with a degradation mediated mainly by the ERAD-proteasome pathway. This latter phenomenon involves a network a proximal ERAD actors, especially proteins of the EDEM family. Modulation of the activity of this network could represent a therapeutic option to counter the oncogenic activity of CALR mutant proteins.

## Supplementary Materials

The following are available online at https://www.mdpi.com/2072-6694/11/12/1921/s1, Figure S1: CALR mutant proteins are faintly expressed in hematopoietic cells, Figure S2: Low level expression of mutant CALR is sufficient to trigger an activation of the STAT pathway and cell proliferation in absence of TPO, Figure S3: CALR mutant proteins are substrates of ERAD-proteasome degradation pathways in hematopoietic cells, Table S1: Characteristics of patients tested for XBP1 splicing.
