# Supplementary material for: The Expression of Myeloproliferative Neoplasm-Associated Calreticulin Variants Depends on the Functionality of ER-Associated Degradation
Source: Cancers (Basel). 2019 Dec 2;11(12):1921. doi: 10.3390/cancers11121921 (PMC6966542; doi:10.3390/cancers11121921)
Supplement: Supplementary file 1 [file cancers-11-01921-s001.pdf]

Supplementarry Materials

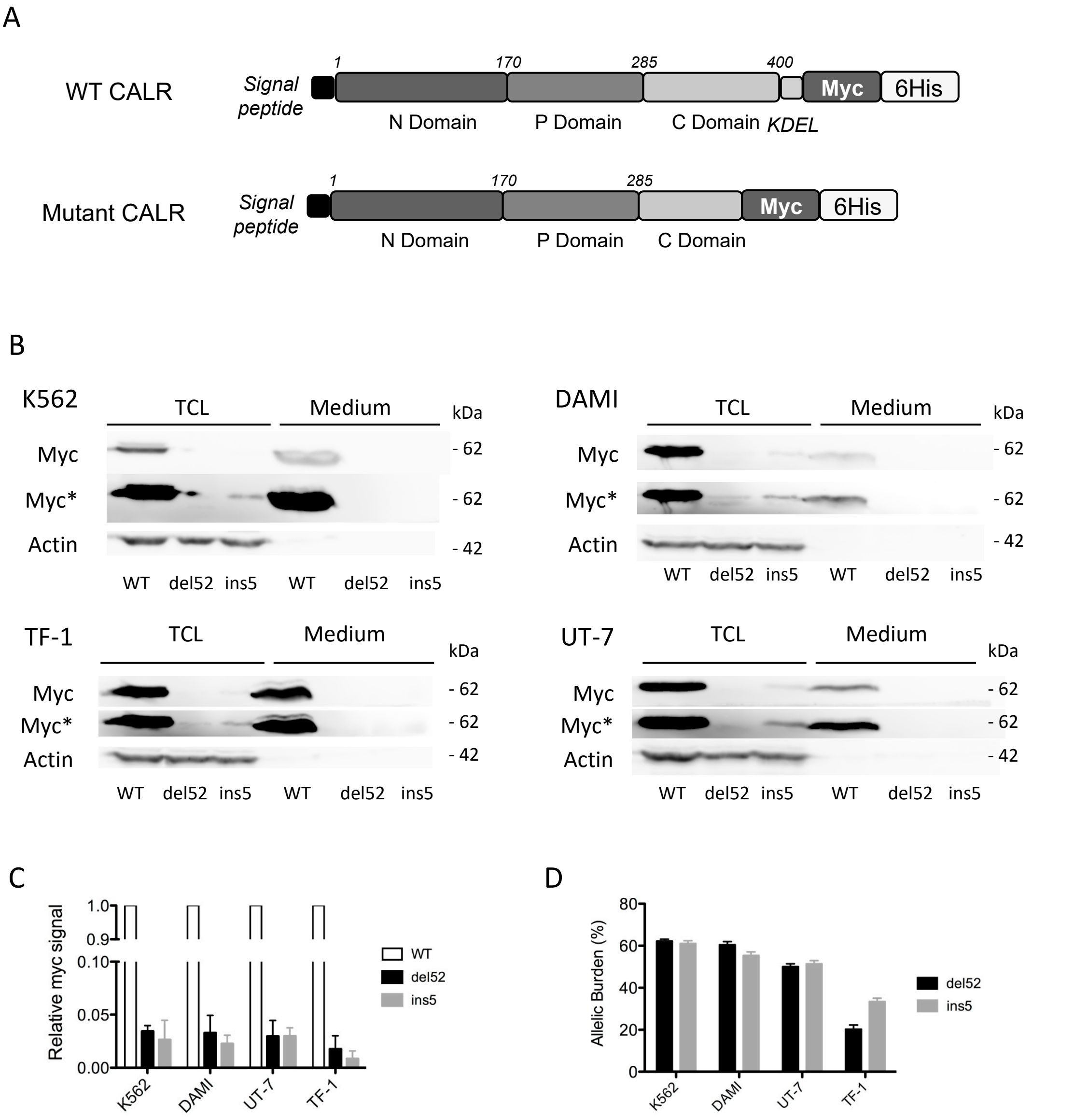

Figure S1. CALR mutant proteins are faintly expressed in hematopoietic cells. Hematopoietic cell lines were transduced with pCW57.1 vector enabling tetracyclin-inducible expression of Myc-6His tagged WT or mutant CALR. (**A**) Schematic representation of Myc-6His tagged CALR. A Myc-6His was added to WT and mutant CALR constructs. The tag was introduced at the C-terminal end of the protein. For the WT CALR, this tag was inserted after the KDEL ER-retention motif. (**B**) Twenty-four hours after induction, cell lysates and supernatants were analyzed by western-blot analysis. Actin serves as a loading control for cell lysates. Exogenous CALR variants were revealed by their Myc epitope. Myc* denotes longer exposure for Myc epitope revelation. These experiments have been performed twice for each cell line. (**C**) Quantification of signals measured on western-blot analysis of total cell lysates performed in (B). Results are expressed as the ratio of Myc to actin signal, normalized on the CALR WT level. The histogram represents the mean ± SEM of 2 independent experiments. (**D**) Quantification of CALR allelic burdens on RNA of indicated

hematopoietic cell lines 24h after induction of transgene expression by doxycycline treatment. Results are expressed as the ratio of mutant CALR to total CALR copy numbers. The histogram represents the mean ± SEM of 3 independent experiments.

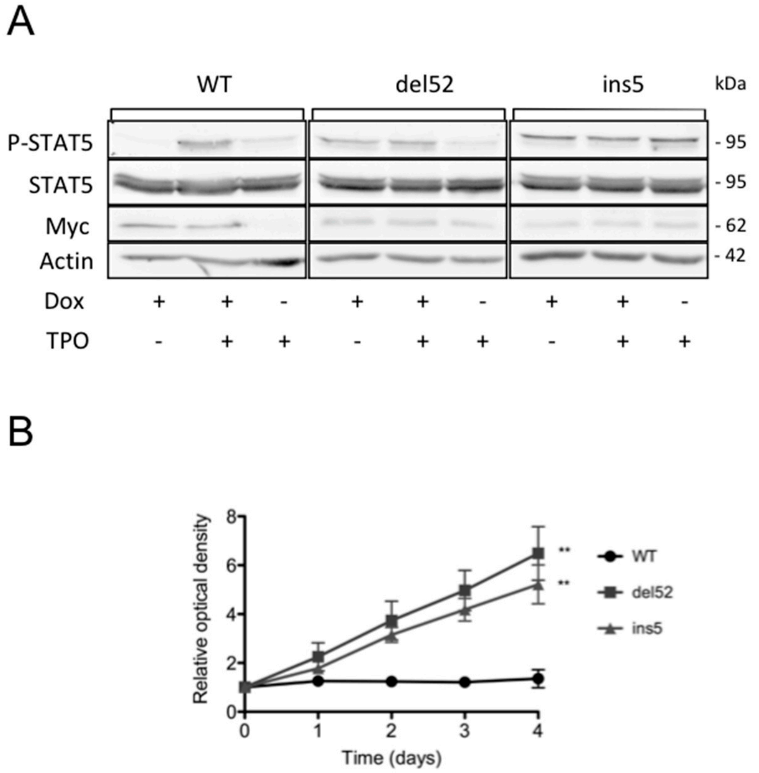

Figure S2. Low level expression of mutant CALR is sufficient to trigger an activation of the STAT pathway and cell proliferation in absence of TPO. UT-7 MPL cells transduced with the tetracyclin-inducible vector pCW57.1 were washed 4 times before being cultured in presence or absence of Doxycyclin (to induce the expression of the CALR transgenes), and in presence or absence of TPO. (**A**) Twenty four hours later, they were lysed, and phosphorylation of STAT5 was assessed by western-blotting. The expression of CALR transgenes was also assessed, but at least for del52 mutant, the band observed correspond to a non-specific signal because it does not present with the expected molecular weight. The absence of specificity of this signal has been checked as shown in Supplemental Figure 3. (**B**) UT-7 MPL cells transduced with the tetracyclin-inducible vector pCW57.1 were washed 4 times before being cultured in presence of Doxycyclin (to induce the expression of the CALR transgenes), but in absence of TPO. Cell proliferation was assessed every day during 4 days. Results are expressed as the optical density normalized on the optical density observed at day 0. The histogram represents the mean ± SEM of 3 independent experiments. ** $p < 0.01$

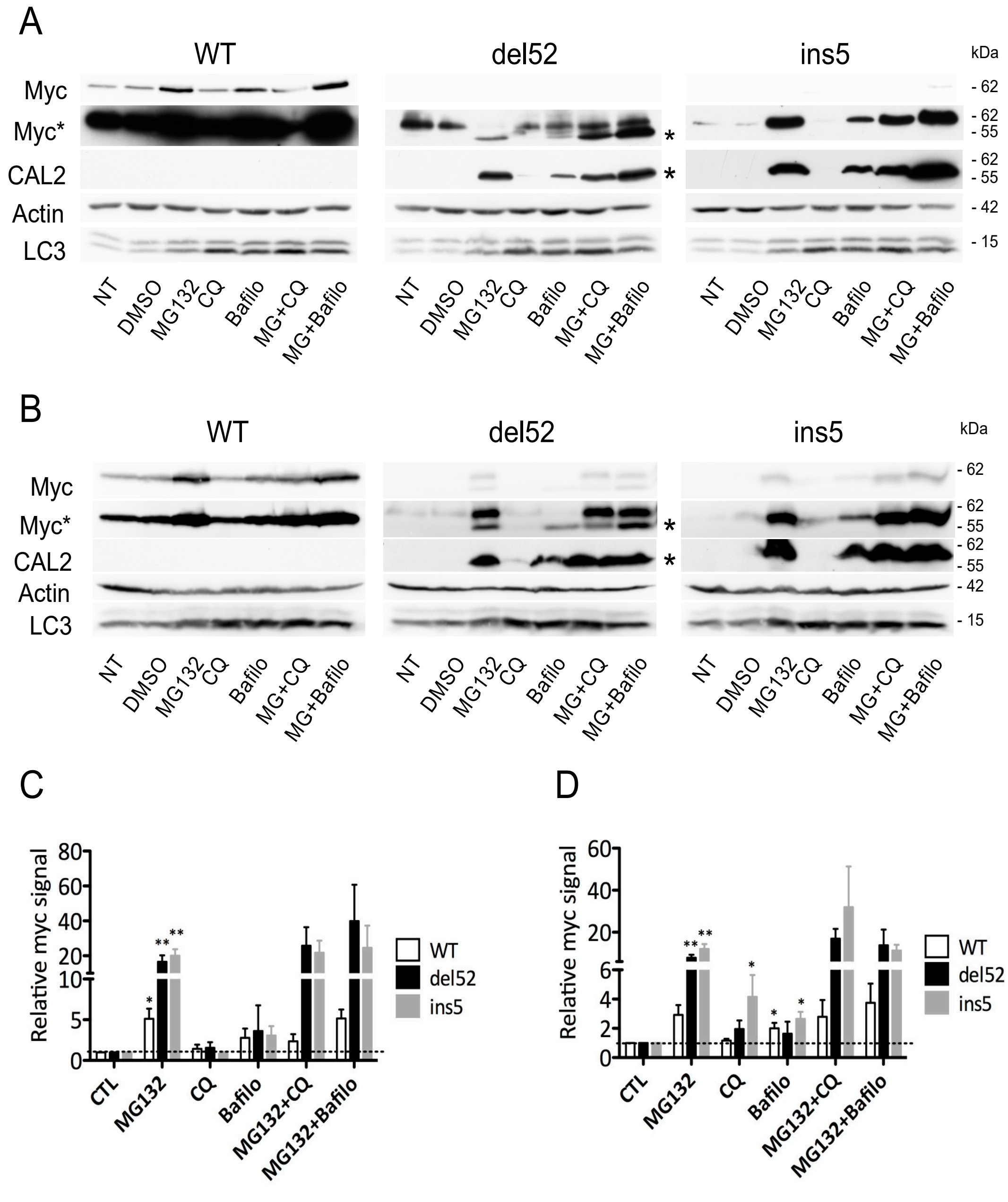

Figure S3: CALR mutant proteins are substrates of ERAD-proteasome degradation pathways in hematopoietic cells. UT-7 MPL (**A**) and DAMI (**B**) cells were transduced with pCW57.1 lentiviral vectors encoding WT or mutant CALR proteins expressing a Myc-6His tag in C-terminus . Twenty four hours after doxycyclin treatment (2µg/mL), cells were treated by MG-132 (10µM), chloroquine (CQ, 50µM), Bafilomycin 1A (Bafilo, 100 nM) or combination for 24h. The transduced CALR proteins were detected using the Myc epitope. CALR mutants were also detected using CAL2 antibody specific to the C-terminal neo-epitope generated by the frameshift mutations. Actin serves as a loading control. Data presented are representative of 7 experiments. Myc* denotes a longer exposure for anti-Myc antibody revelation. DMSO was used as a vehicle control for MG-132 and bafilomycin A1. NT denotes the "Not Treated" condition. For del52 mutants, 2 bands were detected with the anti-Myc antibody, the upper band corresponding to a non-

specific signal and the lower one (marked with *) corresponding to the mutant protein as confirmed by revelation with CAL2 antibody. Expression level of the different forms of CALR proteins in UT-7 MPL (**C**) and DAMI (**D**) was quantified. Results are expressed as a ratio of Myc to actin signal and then normalized against the "not treated" (NT) or "DMSO" condition. Data are expressed as mean ± SEM from 7 experiments. * $p < 0.05$, ** $p < 0.01$.

Table S1. Characteristics of patients tested for XBP1 splicing.

**Patient**	**Diagnosis**	**CALR**		**XBP1 splicing**
		Status	Mutation	
UPN1	ET	P	c.1099_1150del	60.8
UPN2	ET	P	c.1099_1150del	27.2
UPN3	ET	P	c.1154_1155insTTGAC	59.7
UPN4	ET	P	c.1099_1150del	26.0
UPN5	ET	P	c.1099_1150del	44.7
UPN6	PMF	P	c.1099_1150del	13.9
UPN7	ET	P	c.1099_1150del	45.2
UPN8	ET	P	c.1099_1150del	50.7
UPN9	ET	P	c.1154_1155insTTGTC	27.8
UPN10	PMF	P	c.1154_1155insTTGTC	14.2
UPN11	RHL	N	-	35.4
UPN12	RHL	N	-	61.4
UPN13	RHL	N	-	29.4
UPN14	RHL	N	-	42.3
UPN15	RHL	N	-	37.8
UPN16	RHL	N	-	34.4
UPN17	RHL	N	-	34.4
UPN18	RHL	N	-	32.1
UPN19	RHL	N	-	33.8

CALR: calreticulin gene, ET: Essentiel Thrombocythaemia, PMF: Primary Myelofibrosis, RHL: Reactive Hyperleukocytosis and/or thrombocytosis, N: Negative, P: Positive.
